# Supplementary figures and images for: The tuning of tuning: How adaptation influences single cell information transfer
Source: PLoS Comput Biol. 2024 May 13;20(5):e1012043. doi: 10.1371/journal.pcbi.1012043 (PMC11115315; doi:10.1371/journal.pcbi.1012043)

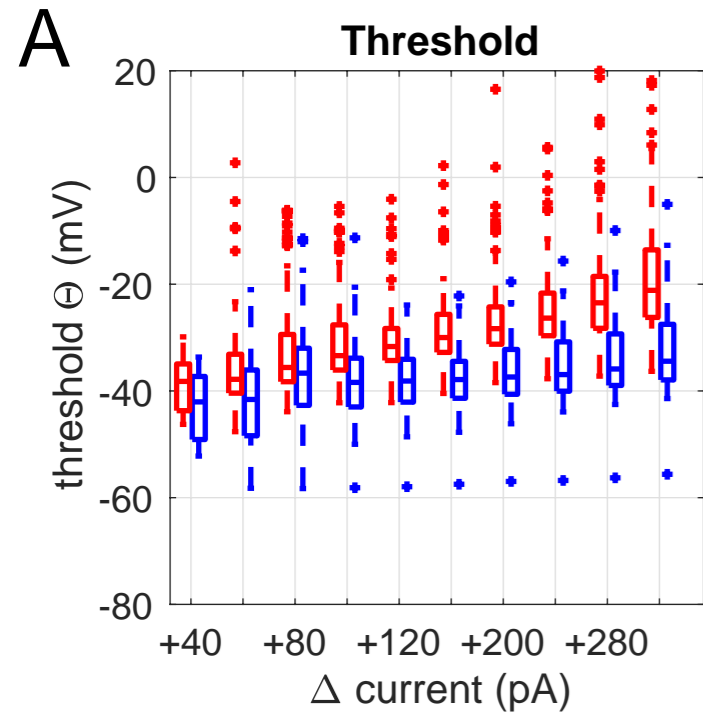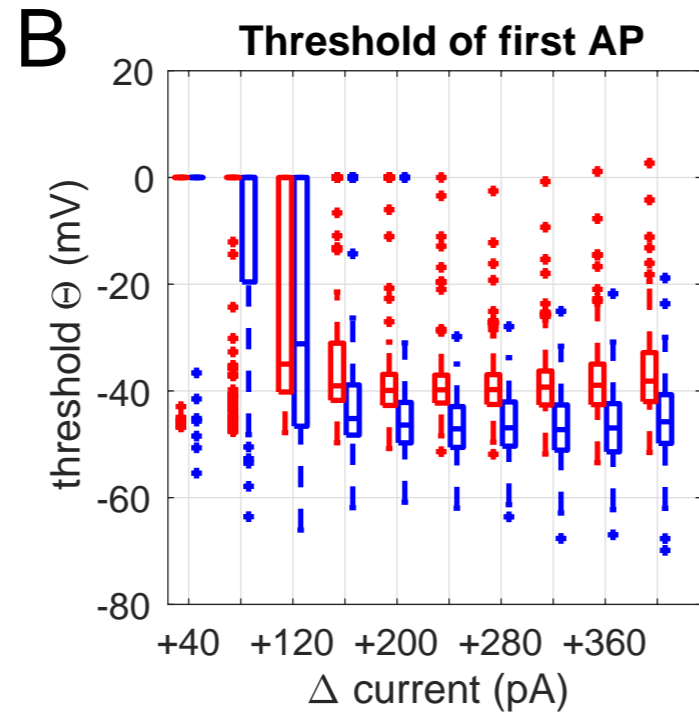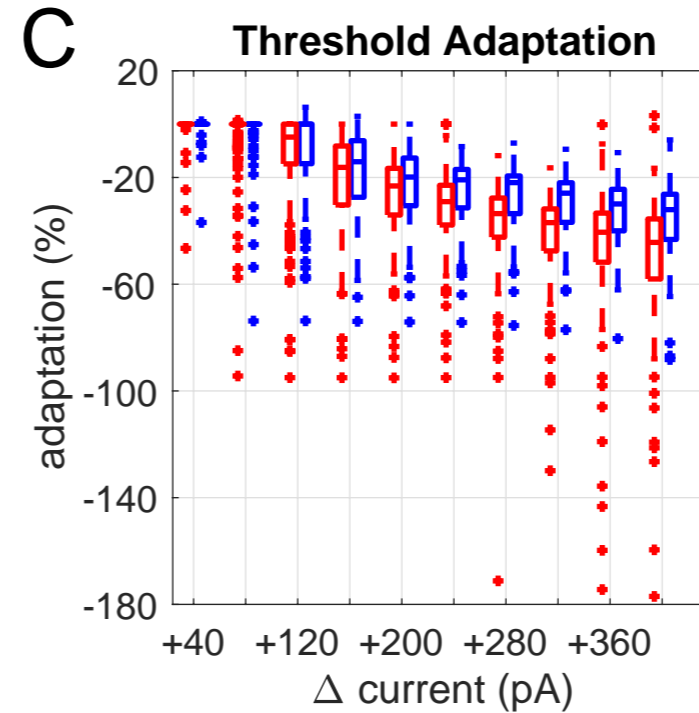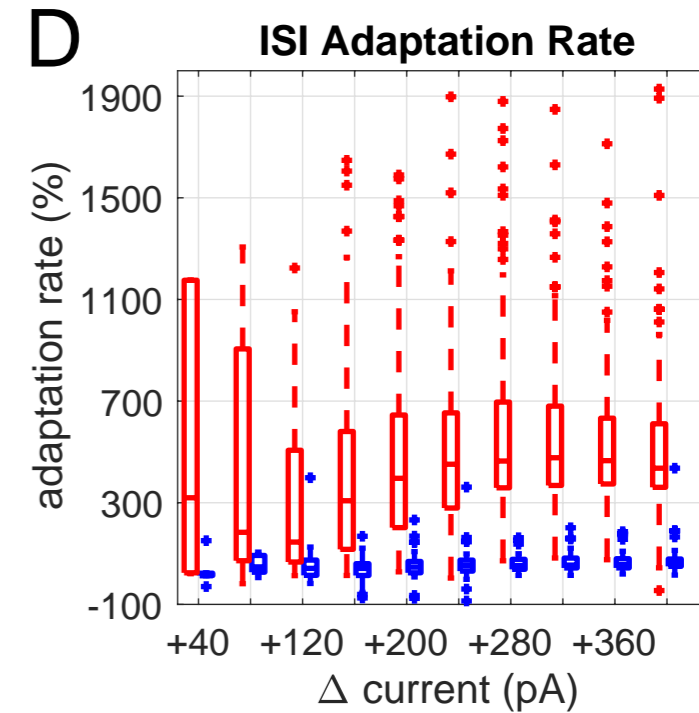

Supplement: S1 Fig — A) Thresholds of all spikes during the step protocol. B) Thresholds of the first spikes after the step current initiation. IC Threshold adaptation: difference in threshold between the first and the last spike of the response. D) Last ISI length relative to the first ISI of the response. Excitatory (red) and inhibitory (blue) neurons. NB Results for significance testing in S1 Table. (PDF) [file pcbi.1012043.s001.pdf]

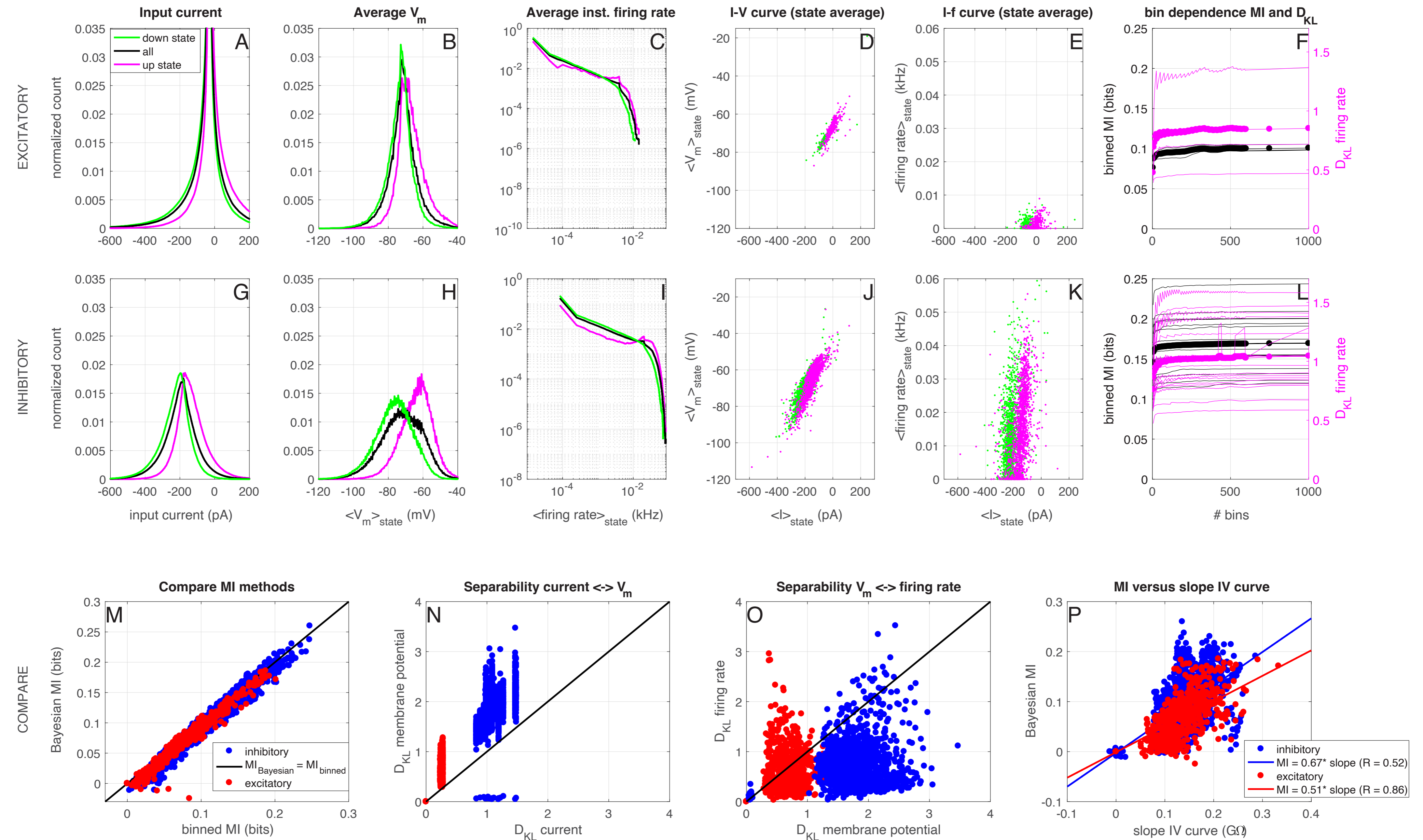

Supplement: S2 Fig — A) Normalized histogram of all (black) input current, the input current when the hidden state was 1 (magenta) and when it was 0 (green) for an excitatory neuron. B) Histogram of the average membrane potential values over each state (i.e. the time between state switches) for all (black) states, the 1 states (magenta) and the 0 states (green) for an excitatory neuron. C) Same as B) but for the instantaneous firing rate (spike train convolved with an exponential kernel, see Materials and methods). D) Average I-V curve: each dot is the average of a single state for the input current (horizontal axis) and membrane potential (vertical axis). A linear curve was fitted for each analysis window to determine the slope of the I − V curve, E) Same as D), but with the instantaneous firing rate on the vertical axis. F) The Mutual information between the hidden state and the instantaneous firing rate (black, left) and the Kullback-Leibler divergence DKL between the distributions of the instantaneous firing rates when the hidden states were 1 or 0 (magenta, right, see panel E) dependence on the number of bins used to bin the instantaneous firing rates (thin lines: single analysis window, lines with dots: average over windows). Based on this panel and panel L, the number of bins was set at 500. G-L) Same as A-F) but for inhibitory neurons. M) Comparison of the two methods to calculate the mutual information between the hidden state and the output spike trains (blue dots: inhibitory neurons, red dots: excitatory neurons). N) The Kullback-Leibler divergence between the histograms of the average over the state when the state was 1 or 0 (i.e. the ‘separability’) of the current (horizontal axis) and membrane potential (vertical axis). Note that for both inhibitory and excitatory neurons the membrane potential has a higher DKL than the input current. O) Same as N), but for the membrane potential and the instantaneous firing rate. P) The relation between the slope of the I − V curve (see p [file pcbi.1012043.s002.pdf]
