# Supplementary material for: The tuning of tuning: How adaptation influences single cell information transfer
Source: PLoS Comput Biol. 2024 May 13;20(5):e1012043. doi: 10.1371/journal.pcbi.1012043 (PMC11115315; doi:10.1371/journal.pcbi.1012043)
Supplement: S1 Table — (DOCX) [file pcbi.1012043.s003.docx]

| **Current Injected** | **Mean AP threshold** | **First AP threshold** | **AP threshold Adaptation Rate** | **Maximum Firing Rate** | **Latency to first AP** |
| --- | --- | --- | --- | --- | --- |
| **+40pA** | d = -0.60  CI [-11.2, 3.7]  p = 0.30 | d = -0.29  CI [-6.3, -0.02]  p = 0.048 | d = -0.056  CI [-1.7, 1.1]  p = 0.70 | d = 0.36  CI [0.26, 2.3]  p = 0.01 | d = 0.2  CI [-101, 136]  p = 0.75 |
| **+80pA** | d = -0.60  CI [-13.6, -0.15]  p = 0.045 | d = -0.15  CI [-8.2, 2.5]  p = 0.3 | d = -0.075  CI [-4.9, 2.7]  p = 0.61 | d = 0.71  CI [3.8, 9.1]  p = 2.4*10^-6^ | d = -0.84  CI [-121, -21]  p = 0.0062 |
| **+120pA** | d = -0.42  CI [-8.5, -0.34]  p = 0.034 | d = -0.066  CI [-7.3, -4.6]  p = 0.65 | d = -0.0055  CI [-5.6, 5.3]  p = 0.97 | d = 0.90  CI [8.1, 15]  p = 3.9*10^-9^ | d = -0.75  CI [-67, -21]  p = 2.1*10^-4^ |
| **+160pA** | d = -0.84  CI [-9.2, -4.2]  p = 4.1*10^-7^ | d = -0.38  CI [-11.1, -1.5]  p = 0.0097 | d = 0.070  CI [-4.1, 6.8]  p = 0.63 | d = 1.4  CI [18, 27]  p = 1.0*10^-19^ | d = -0.74  CI [-44, -18]  p = 6.6*10^-6^ |
| **+200pA** | d = -1.1  CI [-8.9, -5.2]  p = 2.0*10^-12^ | d = -0.65  CI [-11.1,-4.3]  p = 1.2*10^-5^ | d = 0.16  CI [-2.2, 7.7]  p = 0.28 | d = 2.2  CI [31, 40]  p = 8.0*10^-38^ | d = -1.05  CI [-28, -15]  p = 3.0*10^-11^ |
| **+240pA** | d = -1.2  CI [-10.5, -6.4]  p = 9.1*10^-15^ | d = -1.2  CI [-10.3, -6.2]  p = 8.9*10^-14^ | d = 0.32  CI [0.5, 9.1]  p = 0.028 | d = 3.3  CI [42, 51]  p = 4.6*10^-59^ | d = -1.03  CI [-21, -11]  p = 3.7*10^-11^ |
| **+280pA** | d = -1.2  CI [-11.4, -7.1]  p = 1.8*10^-15^ | d = -1.2  CI [-9.3, -5.7]  p = 4.7*10^-14^ | d = 0.47  CI [3.8, 16.4]  p = 0.0017 | d = 4.3  CI [51, 58]  p = 4.4*10^-78^ | d = -1.4  CI [-12.4, -8.3]  p = 3.5*10^-19^ |
| **+320pA** | d = -1.3  CI [-12.8, -8.1]  p = 1.1*10^-15^ | d = -1.2  CI [-10.2, -6.2]  p = 1.6*10^-14^ | d = 0.30  CI [0.46, 30.2]  p = 0.043 | d = 4.9  CI [58, 65]  p = 2.4*10^-88^ | d = -1.5  CI [-9.2, -6.2]  p = 5.1*10^-21^ |
| **+360pA** | d = -1.3  CI [-14.6, -9.1]  p = 2.4*10^-15^ | d = -1.2  CI [-11.0, -6.6]  p = 1.7*10^-13^ | d = 0.14  CI [-9.2, 27.1]  p = 0.33 | d = 5.4  CI [64, 71]  p = 2.02*10^-96^ | d = -1.5  CI [-7.1, -4.8]  p = 1.3*10^-20^ |
| **+400pA** | d = -1.2  CI [-17.1, -10.4]  p = 2.7*10^-14^ | d = -1.1  CI [-12.0, -7.1]  p = 4.9*10^-13^ | d = 0.21  CI [-5.0, 33]  p = 0.15 | d = 5.6  CI [68, 76]  p = 1.7*10^-99^ | d = -1.5  CI [-5.6, -3.7]  p = 5.8*10^-20^ |

| **Current Injected** | **Mean AHP**  **Peak**  **Amplitude** | **Mean AP**  **Half-Width** | **ISI**  **Adaptation**  **Rate** | **ISI**  **Mean** | **ISI**  **Minimum** |
| --- | --- | --- | --- | --- | --- |
| **+40pA** | d = -0.18  CI [-11, 3.7]  p = 0.29 | d = -1.2  CI [-0.70, -0.08]  p = 0.016 | d = -0.65  CI [-2.1*10^3^, 526]  p = 0.7020 | d = -0.75  CI [-77, 21]  p = 0.23 | d = 0.079  CI [-60, 68]  p = 0.88 |
| **+80pA** | d = 0.02  CI [-13, -0.15]  p = 0.045 | d = -1.8  CI [-0.86, -0.43]  p = 1.7*10^-7^ | d = -0.65  CI[-1.1*10^3^, -13]  p = 0.044 | d = -1.5  CI [-110, -47]  p = 8.1*10^-6^ | d = -0.81  CI [-84, -11]  p = 0.011 |
| **+120pA** | d = 0.04  CI [-8.5, -0.34]  p = 0.034 | d = -2.02  CI [-0.9, -0.6]  p = 1.9*10^-18^ | d = -0.63  CI [-512, -90]  p = 0.0056 | d = -1.2  CI [-87, -42]  p = 1.3*10^-7^ | d = -0.69  CI [-70, -16]  p = 0.0016 |
| **+160pA** | d = 0.01  CI [-9.2, -4.1]  p = 4.1*10^-7^ | d = -2.1  CI [-0.94, -0.69]  p = 9.2*10^-29^ | d = -1.2  CI [-481, -272]  p = 3.1*10^-11^ | d = -1.3  CI [-64, -38]  p = 1.8*10^-12^ | d = -0.53  CI [-38, -8.9]  p = 0.0018 |
| **+200pA** | d = 0.11  CI [-8.9, -5.2]  p = 2.0*10^-12^ | d = -2.2  CI [-1.04, -0.80]  p = 7.0*10^-35^ | d = -1.3  CI [-521, -330]  p = 6.3*10^-16^ | d = -1.9  CI [-61, -44]  p = 2.6*10^-27^ | d = -0.57  CI [-25, -7.7]  p = 2.5*10^-4^ |
| **+240pA** | d = 0.14  CI [-10, -6.4]  p = 9.1*10^-15^ | d = -2.1  CI [-1.1, -0.86]  p = 9.1*10^-34^ | d = -1.4  CI [-559, -364]  p = 1.0*10^-17^ | d = -1.4  CI [-53, -35]  p = 5.8*10^-19^ | d = -0.29  CI [-17, 0.031]  p = 0.0508 |
| **+280pA** | d = 0.24  CI [-11, -7.2]  p = 1.8*10^-15^ | d = -2.0  CI [-1.2, -0.95]  p = 1.3*10^-31^ | d = -1.7  CI [-587, -417]  p = 8.3*10^-25^ | d = -2.5  CI [-43, -34]  p = 6.6*10^-43^ | d = -0.46  CI [-6.8, -1.5]  p = 0.0021 |
| **+320pA** | d = 0.36  CI [-12, 8.1]  p = 1.1*10^-15^ | d = -1.9  CI [-1.4, -1.0]  p = 3.0*10^-29^ | d = -1.7  CI [-591, -419]  p = 1.3*10^-24^ | d = -2.8  CI [-37, -30]  p = 2.3*10^-50^ | d = -0.59  CI [-4.5, -1.6]  p = 7.3*10^-5^ |
| **+360pA** | d = 0.49  CI [-14.6, -9.1]  p = 2.3*10^-15^ | d = -1.9  CI [-1.5, -1.1]  p = 1.4*10^-28^ | d = -1.7  CI [-570, -406]  p = 2.9*10^-25^ | d = -3.1  CI [-34, -29]  p = 2.7*10^-56^ | d = -0.77  CI [-3.9, -1.8]  p = 3.9*10^-7^ |
| **+400pA** | d = 0.62  CI [-17, -10]  p = 2.7*10^-14^ | d = -1.8  CI [-1.7, -1.3]  p = 2.7*10^-26^ | d = -1.7  CI [-534, -376]  p = 7.9*10^-24^ | d = -3.1  CI [-32, -26]  p = 9.9*10^-55^ | d = -0.91  CI [-3.9, -2.0]  p = 3.7*10^-9^ |

P = p-value (according to 2-sample t-test)

d = Cohen’s d (Measure of effect power); positive means inhibitory has a higher value.

CI = 95% Confidence Interval (for effect power)

**Supplementary Table S1**: Statistical tests of the comparison between excitatory and inhibitory neurons in the current clamp step protocol.
