# Supplementary material for: The tuning of tuning: How adaptation influences single cell information transfer
Source: PLoS Comput Biol. 2024 May 13;20(5):e1012043. doi: 10.1371/journal.pcbi.1012043 (PMC11115315; doi:10.1371/journal.pcbi.1012043)
Supplement: S2 Table — P-values were compared to a threshold of 5% / 6 groups = 29 0.83% (Bonferroni correction). (DOCX) [file pcbi.1012043.s004.docx]

|  | N inh | N exc | mean inh | mean exc | h KS test | p KS test | KS stat | h WR test | p WR test | Cliff's Delta |
| --- | --- | --- | --- | --- | --- | --- | --- | --- | --- | --- |
| # spikes per up state | 1404 | 560 | 0.99 | 1.22 | 0 | 0.047 | 0.068 | 0 | 0.59 | 2.52 |
| # spikes per down state | 1404 | 560 | 0.15 | 0.49 | 1 | 5.8e-10 | 0.16 | 1 | 9.4e-10 | 2.33 |
| firing rate up (Hz) | 1404 | 560 | 12.8 | 3.52 | 1 | 7e-155 | 0.66 | 1 | 7e-163 | 3.29 |
| firing rate down (Hz) | 1404 | 560 | 1.04 | 0.63 | 1 | 1.4e-43 | 0.35 | 1 | 8.1e-48 | 2.93 |
| normalized firing rate up | 1404 | 560 | 0.64 | 0.79 | 1 | 0.0015 | 0.094 | 0 | 0.0579 | 2.45 |
| normalized firing rate down | 1404 | 560 | 0.052 | 0.138 | 1 | 3.0e-8 | 0.149 | 1 | 1.9e-8 | 2.34 |

**Supplementary** **Table S2**: Statistical tests of the comparison between excitatory and inhibitory neurons in the frozen noise protocol (see main text Fig. 5). P-values were compared to a threshold of 5% / 6 groups = 0.83 % (Bonferroni correction).
