# Supplementary material for: The tuning of tuning: How adaptation influences single cell information transfer
Source: PLoS Comput Biol. 2024 May 13;20(5):e1012043. doi: 10.1371/journal.pcbi.1012043 (PMC11115315; doi:10.1371/journal.pcbi.1012043)
Supplement: S3 Table — P-values were compared to a threshold 34 of 5% / 6 groups = 0.83% (Bonferroni correction). (DOCX) [file pcbi.1012043.s005.docx]

|  | N inh control | mean inh  control | h KS test | p KS test | KS stat | h WR test | p WR test | Cliff's Delta |
| --- | --- | --- | --- | --- | --- | --- | --- | --- |
| # spikes per up state | 33 | 3.77 | 1 | 1.0e-18 | 0.80 | 1 | 2.5e-16 | 0.91 |
| # spikes per down state | 33 | 2.62 | 1 | 1.0e-19 | 0.82 | 1 | 3.0e-16 | 0.83 |
| firing rate up (Hz) | 33 | 10.87 | 1 | 1.5e-18 | 0.79 | 1 | 2.5e-16 | 0.91 |
| firing rate down (Hz) | 33 | 3.43 | 1 | 1.0e-19 | 0.82 | 1 | 7.3e-16 | 0.89 |
| normalized firing rate up | 33 | 2.62 | 1 | 1.5e-18 | 0.80 | 1 | 2.5e-16 | 0.91 |
| normalized firing rate down | 33 | 0.86 | 1 | 1.0e-19 | 0.82 | 1 | 7.3e-16 | 0.89 |

**Supplementary** **Table S3**: Statistical tests of the comparison between excitatory and inhibitory neurons receiving the control frozen noise stimulus (see main text Fig. 5). P-values were compared to a threshold of 5% / 6 groups = 0.83 % (Bonferroni correction).
