# Supplementary material for: The tuning of tuning: How adaptation influences single cell information transfer
Source: PLoS Comput Biol. 2024 May 13;20(5):e1012043. doi: 10.1371/journal.pcbi.1012043 (PMC11115315; doi:10.1371/journal.pcbi.1012043)
Supplement: Attachment — Submitted filename: Response_reviewers.pdf [file pcbi.1012043.s006.pdf]

DONDERS INSTITUTE

Dr Fleur Zeldenrust
Associate professor in Computational Neuroscience
Leader of theme 4: Natural Computing and Neurotechnology

Heyendaalseweg 135
6525 HJ Nijmegen
The Netherlands

fleur.zeldenrust@donders.ru.nl
fleurzeldenrust.nl

To: PLOS Computational Biology

Nijmegen, February 14th 2024

Dear Dr. Graham,

Hereby, we would like to submit the attached revised manuscript for review. We would like to thank the reviewers for their thorough reading and constructive feedback. We have performed the alternative analysis suggested by reviewer 2, and added this to the Methods section. Moreover, we included the results in Supplemental Figure 2. We also extended the discussion section considerably, to discuss the limitations of the experimental setup we used and what that means for the interpretation of the coding the neurons perform. We also corrected a small error in Figure 1. We believe that with this, the manuscript has improved considerably.

Please find a point-by-point summary of how we addressed the reviewer comments below.

Best regards,

Fleur Zeldenrust
Niccolò Calcini
Xuan Yan
Ate Bijlsma
Tansu Celikel

PS Note that the doi for the data repository given in the paper does not work yet, as the repository doi can only be finalized upon publication. However, there is a special (updated) 'reviewer access' to the database: https://data.ru.nl/login/reviewer-399946720/6VWMTUBIFDPQI4A3S53YUNABVCE5S6ACCMJCCIY

Reviewer #1:

The paper describes a combination of modeling and ex-vitro experiments (in mouse barrel cortex) on excitatory and inhibitory neurons in mouse barrel cortex. The goal of the work is to elucidate the effect on information transmission of certain adaptive mechanisms in the neuron’s spike generating mechanism. This work relates to an important issue in neuroscience: How do individual neurons change some of their physiological properties to optimize signal transfer, as measured by Shannon information. The combination of actual experiments with detailed simulation is nice, since it allows the authors to explore a number of well-known properties of the cells that are not amenable to direct experimental manipulation. Both the analysis and the experiments are described in sufficient detail, and seem to be carried out very well.
We thank the reviewer for their support and constructive feedback.

Major comments

Overall, I do not have any issues with how the project was executed and with the results that were obtained in both experiment and simulation. I do, however, have a serious issue with the overall interpretation of the results in a wider context: Throughout the paper the authors quantify mutual information between a source (a 2-state Markov model) and a receiver (both the output of an Artificial Neural Network (ANN), or the spiking output of a neuron using the ANN signal as its input). The methods for analyzing this situation were worked out in reference 15, and they look correct to me. The authors claim correctly that this method is efficient, in the sense that one can get reliable estimates in relatively short time (order of 5 minutes), as compared to more exhaustive methods that may take an hour or more of experiment time. I can appreciate that it is crucial to use fast methods in an experiment where the cells cannot be held for very long.
However, there is an important downside to the analysis as well, which is nowhere discussed: That is that the source is likely to be an impoverished representation of the signals that are representative of the whisker system. The main information measures presented here quantify mutual information between the hidden Markov state on the one hand and the neuron’s firing rate on the other. In other words, what is measured is the relation between a two-state system and a spike count. Spike timing is not considered as an information carrier, and neither is the dynamic structure of more-or-less natural inputs.
We agree with the reviewer's analysis of the method, and that the method uses a reduction in entropy of the input (a binary hidden state) to speed up the information calculation. Also, we indeed do not include so-called 'pattern codes' or 'phase codes', although the integration of the log-likelihood L does include the timing of individual spikes. We have added paragraphs in the discussion about the limitations of our method.

Over the last few decades, it has become clear that in many sensory systems, spike timing plays an important role in information transmission (indeed, many of the key publications are cited in the present paper). Over roughly that same time frame a large body of literature has developed which points to the whisker system, and neurons in barrel cortex as sparse encoders, which can be very finely tuned to temporal structure (e.g. the encoding of textures as sensed by the whiskers). None of that literature is cited here. So, while I understand the necessity of using a fast and efficient method for measuring information transmission, it is incumbent upon the authors to also discuss its limitations in the more general context of neural information transmission, in particular in the barrel cortex, but perhaps also in other modalities.
Thank you for the suggestion, we have added a paragraph in the discussion about this.

They should, I think, also acknowledge that the low spike rate, and the low information throughput of the excitatory cells that they report might well be an artefact of their particular methods (case in point: because of the different firing rates of excitatory and inhibitory cells, the Markov switching rates are adjusted for each cell type: That already shows that the method lacks generality). Conceivably, for example, a full analysis might prove that the excitatory cells are highly selective to particular stimulus features, and may therefore signal a lot of information per spike.
We do not believe that the low spike rate of our excitatory cells is an artefact, this is a well documented property of barrel cortex (see https://pubmed.ncbi.nlm.nih.gov/15064767/ for excitatory neurons, https://pubmed.ncbi.nlm.nih.gov/32027647/ for inhibitory neurons and https://pubmed.ncbi.nlm.nih.gov/22579264/ for a review). We added this to the discussion.
It might indeed be that excitatory cells are very selective for a specific feature, and therefore lossy in general. This is also suggested in figure 11, which shows that excitatory cells are more selective for different features, and inhibitory cells more uniform in their response. We have added this also to the discussion.

I do not mean to suggest that the authors perform a whole new set of (potentially impossible) experiments, but I do think it is important to address carefully the limitations of their particular analysis.
We hope that with the aforementioned additions these are now addressed sufficiently.

Minor points - all adapted as suggested.

Line 50: information processing rate -> information transmission rate?
Line 62: these to forms -> these two forms
Legend to Fig 1E: Contrary to the test in the legend, it looks to me that the red cloud of AP half widths of the excitatory cells overlaps completely with those of the inhibitory cells (and the shortest red ones are shorter than the shortest blue ones). But that is inconsistent with panel I of the same figure. Am I missing something?
You are absolutely right! We made a small error in the plotting of this figure, which resulted in an axis inversion for panel E. We have corrected this (we have re-done all our analysis just to be sure, but it was indeed just a plotting mistake).
Line 202: and, and -> and
Eq. 8: It is not clear how to interpret the smoothing parameter lambda: IN the equation it looks like a multiplicative factor, but that can't be true. Does it represent the width of some convolution filter? Please explain. There was a plus sign missing from the equation, we have corrected this
Line 373: to entangle -> to disentangle
Fig 6: The right box contains a legend with valued of tau_adap: What is tau_adap, how is it related to the tau values in Eqs 14 and 15? Tau_adap is either tau_w or tau_theta, this is made explicit
Fig 10: Should the units on the horizontal axes of panels C and D be milliseconds^-1 (that is, not seconds^-1 as stated). From the typical values of g_m and C_m one would get time constants of millisecond range - indeed, corrected
Line 666: What is "information compression" in this context? - reformulated
Fig. 11: Panels A, C, E: the STA's are significantly anti-causal. I assume that this is due to some smoothing steps somewhere in the chain of operations, but it also seems to contradict the statement that the stimuli are whitened. Text to panel B: Should "blue line" be replaced by "red line"? The acausal part (i.e. the decay after the spike time) is mostly caused by the autocorrelation of the input (i.e. because of the convolution of the spikes with the exponential kernel with a time constant of 5 ms). The text to panel B was corrected and the anticausal part explained in the results.

Reviewer #2:

This paper addresses an interesting question, the influence of different aspects of adaptation on the ability of neurons to transmit information about their inputs, with a comparison between different neuronal cell types as well as models. The approach here is one that I find rather convoluted and strongly influenced by a specific interpretation of neural firing advanced in previous work by members of this group. It is a very specific and not clearly correct version of information that only pertains to the ability to discriminate two mean input levels in the presence of noise, an ability that can be influenced by internal neuron dynamics in many more ways that are considered here. While I appreciate the significant work that has been put into this analysis, the difficulties in this logic create significant difficulty in interpreting these hardwon results.
We thank the reviewer for their constructive feedback. We hope that with the extra suggested analysis and added paragraphs in the discussion, the interpretation is now clear and the limitations of the method more explicit.

The core of the approach is based on the influential and interesting work by Sophie Deneve and colleagues, including the first author of this paper, who postulated that neural systems may be performing a sort of inference on their inputs, and that their firing properties may be governed by the dynamics of this inference. As a toy problem to illustrate this idea, this previous work proposed a situation in which a system may be faced with signals that may come from one of two sources, each with a certain mean that is obscured by noise. In this case she derived equations for the time-varying estimate of the log-odds of the input being drawn from one or the other of these distributions and related the properties of the computations to biophysical properties of neurons. This idea that an underlying process of ongoing inference in the presence of noise is a mechanism of determining the timing of spikes is very creative: but it is simply an interpretation of the essential mechanism that drives a single neuron to fire. Rather, the timing of spikes of single neurons are governed by detailed temporal fluctuations in the time-varying current that they receive, with no intrinsic meaning needed to be ascribed to "noise" or "signal. For that reason, many previous approaches to quantifying information transmission in neural systems have used complex time-varying inputs and devised methods of computing information, including by giving repeated inputs to estimate noise entropy. In this paper, however, an alternative method is used that takes its inspiration from the "Bayesian neuron" concept. Here one drives the neuron with an input with a "hidden state" that switches between two values, and reduces the evaluation of information to that of computing the information that the firing rate carries about the value of the hidden state, ie a maximum of one bit.
We agree with this analysis. Small comment: the hidden state has an entropy of slightly less than one bit, as it is in the 'off' state more often than in the 'on' state. This has been made explicit in the Methods section.

This on its own is artificial, and a vast simplification of neuronal response, but OK. The signal is generated as a sum of 1000 Poisson processes for which the hidden state serves as the rate; each point process is then convolved with an exponential kernel and summed.
We agree with the reviewer that in ex-vivo experiments, input is often artificial, because the exact spatiotemporal distribution of input to a neuron is often not known. Traditionally, either 'step-and-hold' or white noise inputs are used. In that sense, Poisson processes convolved with an exponential kernel are equally or even more naturalistic than the classical inputs. Also, many of the other types of analysis (i.e. reverse correlation analysis) remain possible. The fact that the noisy, fluctuating input is generated by a binary hidden state is an addition that makes the mutual information calculation easier. A dynamic clamp (in which a conductance rather than a current is injected) process would be more realistic, and we are currently developing a protocol for this. However, this is beyond the scope of the current experiments. We have added this consideration to the discussion.

Thus, the time-varying hidden state is transformed into something close to a white noise process with a local mean and standard deviation. One can then ask how well can one determine the mean from a chunk of signal in the presence of this noise; and then, how does driving a spiking neuron with this input, re-representing it in spiking output, affect this level of discriminability. While in general one could have considered discriminating two mean values of rate, here the readout is taken to be spikes or no spikes, ie requiring the system to be in a regime in which the DC level of the lower state is subthreshold for spiking. Because of this, the information should be limited by several things. The most important effect should be the nature of the f-I curve (firing rate vs mean current) for the neuron: how steep it is, how far apart are the input DC values, governing how well separated for these chosen values the mean firing rate of the neuron will be, how the firing rate as a function of the mean current is influenced by background noise (Chance and Abbott; Higgs and Spain), how the variance of the effective background noise changes this relationship. One should also discuss the choice of the time bin in

which to probe the firing, and how this interacts with the neuron's refractory period. None of these issues are explicitly discussed.
We thank the reviewer for this valuable suggestion.
Indeed, how the mean and standard deviation of the input differ between the two hidden states depends on how the rates qon and qoff of the ANN are chosen. If qon and qoff are chosen from different distributions, for instance with a different mean, this indeed results in a difference in average input current between the two states. In our case, qon and qoff are chosen from the same distribution. For clarity, we have added these distributions to the new Supplemental Figure 2 (A and G).
We agree with the reviewer that there is a relationship between the neuron's effective working range (i.e. the f-I curve), the dynamic range of the input, and the transferred information. What makes this extra difficult is that the working range of a neuron is typically not fixed but depends on the characteristics of the input (ie its power spectrum, as most neurons are either low-pass filters and/or have a resonant frequency, its amplitude, its mean, and more, as indeed discussed in amongst others the suggested papers). Indeed, an f-I curve might be completely different for a 'noisy' input current than for a step-and-hold input current, and the same is true for an I-V curve. How these (dynamic and heterogeneous) neuron properties interact with information transfer is exactly the object of investigation of this paper. We already assessed the dynamic range by looking at the (steepness of the) I-V curves of model (figure 8 and 9) and recorded (figure 10) neurons. As the reviewer suggests, we added a mean input current - membrane potential and mean input current - output frequency curves on an state-by-state basis (i.e. between state switches) in Supplemental Figure 2 (D, E and J, K) and correlated the slope of the IV curve with the mutual information between the hidden state and the firing rate (Supplemental Figure 2 P). This leads us to the same conclusion as figure 10: A greater resistance / smaller conductance increases the information transfer.
We added a discussion about the working range and the suggested citations to the discussion.
Finally, when performing the alternative calculation of the MI as suggested, we used an exponential kernel to make a time series of the instantaneous firing rate, of which the decay time (50 or 250 ms) was much longer than the time bin with which both experiments and simulations were performed (sampling rate: 20 kHz). Therefore, the choice of time bin should not affect the value of the MI. However, the number of bins in the histograms does influence the Kullback-Leibler divergence an mutual information. This was made explicit in Supplemental Figure 2 F and L. We hope that with this alternative calculation, the reviewer is assured that both methods are valid.

The issue in which the paper invests most analytical machinery is that of the time dependence of the switches. This is important as it will interact with the temporal integration time of the neuron. In general, it will take some time following a switch in the input DC before the steady state firing rate for the new input is reached, which can be due to the neuron's intrinsic filtering properties, or to internal dynamics of the neuron that change in response to the change in DC input. Thus, the ability to discriminate a change will depend on how rapidly the hidden state is changing. If the state varies so rapidly that the receiving neuron does not reach steady state, it will be difficult to make the necessary discrimination. Thus generally over some fraction of the time in which the input is held at a given hidden state, and again depending on the nature of the processing of that neuron, the ability to discriminate the DC level will be nonoptimal.
We agree with this analysis. This delay in reaching the steady state depends on both the firing rates in the SPNN (see figure 2B) and the integration time(s) of the neuron (see figure 10).

The information calculation is done in a way that to me was confusing and is very reliant on the "Bayesian neuron" concept. To evaluate the information in the input about the hidden state, one could simply form the distributions of input (averaged, presumably, over bins of a chosen size) conditioned on times at which the state was high and in which it was low, and compute the mutual information as the entropy of the total input distribution minus the appropriately weighted entropy of the conditional distributions. Given the simple nature of the input, this mutual information could probably be computed analytically. One would then do the same for the firing rate. This would give the MI between state and input, and the MI between spikes and input, from which one could compute the fraction.
We performed the suggested alternative calculation of the mutual information and compared the results (Supplementary figure S2 and the paragraph explaining this analysis in the Materials and Methods section). The results are quite similar (see Fig S2, panel M). Because of that, we kept the MI calculation throughout the paper as is, but added a paragraph in the Materials and Methods section explaining the two ways of computing the MI, and their respective advantages and disadvantages.

The rate of the switches between high and low will generally always matter to this information calculation because of the neuron's multiple time-constant filtering and processing properties (whether it is intrinsically an integrator or differentiator, whether it is fast-filtering or slow, whether there are slow time constants of

adaptation that depend on the rate of switching), putting different fractions of the firing rate into a transient "ambiguous" zone depending on this switching rate, so I'd be inclined to plot the information out as a function of that switching time.
We agree with the reviewer that the filtering properties of neurons essentially determine its information transfer. Actually, we are currently starting a collaboration with a research group in Berlin to see whether we can determine whether a neuron is type 1 or 2 (integrator or resonator) based on our measurements, and how that influences information transfer. However, this is for now beyond the scope of the current paper. Here, we aimed to investigate the effects of a few of the basic properties of neurons (IV curve slope and adaptation) on information transfer. However, of course there are many other neuron properties that probably play an important role. This is shown by the fact that we can never make the information transfer of the expIF neuron as high as that of the Bayesian Neuron and of the experimentally recorded neurons. We have added an extensive discussion about the limitations of our method to the discussion.

As an aside-- it's not obvious that a spiking neuron necessarily loses information about this mean relative to the input itself. For example, as alluded to above, a neuron that acts as a fractional differentiator in a range of timescales that are close to the switching rate (ie is less sensitive to the noise but rather responds to the DC) could even highlight the moment of state transition relative to the unfiltered original input, as well as retain information about the steady state (Lundstrom et al, 2007).
Indeed, one could imagine a neuron that just responds to an up-switch or down-switch. We added this paper and several forms neural responses could take to the discussion section.

Overall the approach taken in the paper relative to the simpler version outlined above seems like an overly model-driven approach, in which clarity about the role of different mechanisms was lost.
We hope that with the added analysis, as the reviewer suggested, we can convince the reviewer that the way the mutual information is calculated is correct. As for the experimental set-up, all the types of analysis that one can typically do with a white noise input (i.e. reverse correlation analysis) can still be done (Fig 11). All our setup does is add an easy mutual information calculation.

A few specific comments.

It seems an exaggeration to call the stimulus construction procedure an "ANN". It is simply the sum of many independent Poisson processes, filtered with synaptic time course. Calling it an ANN implies that there is connectivity or meaningful dynamics generated by these simulated neurons.
We have called it this, because we experienced that when presenting the method sometimes colleagues found it confusing to see what was part of the experiment (ie 'real' brain) and what was simulated. Changed it to Simulated Poisson Neural Network (SPNN) for clarity.
It is unclear and not explained why the input has to be "frozen" noise. Is this to hold the relative times spent in the two states and in the transitions between them fixed across experiments?
Indeed, an input current that is a new realization of the same stochastic process (generation of hidden state and the generation of the resulting input current) could have been generated for each trial. We have chosen not to do that, because of 2 reasons:

1. an experimental setup constraint: the current had to be generated offline and loaded onto the setup, which would have made the experiments take a lot longer
2. The experiments were in many neurons also repeated with different neuromodulators (not presented here, but see Yan, X., Calcini, N., Safavi, P., Ak, A., Kole, K., Zeldenrust, F., & Celikel, Tansu. (2022). A whole-cell recording database of neuromodulatory action in the adult neocortex. *bioRxiv*. https://doi.org/10.1101/2022.01.12.476007). For proper comparisons between aCSF and neuromodulator trials (ie omitted or added spikes for the same input), the input current needed to be frozen.

We have explained this now in the methods section.
What is the meaning or relevance of the mean current across the entire trial, Fig C and D?
It is $I_{baseline}$, see table 1, which is different for each neuron. We clarified this in the caption.

The input is scaled to sit at a particular point on the neuron's I-V curve. This should be more clearly explained and justified in the light of the above discussion.
We added a paragraph in the discussion about the gain modulation and the I-V curve. We also made this explicit in the Materials and Methods.

The calculation of the mutual information between hidden state and input should be simple and does not seem to

need the formalism of the log odds (see above); this section is extremely hard to follow. If x = state and y = other variable (input or rate), in the approach taken above, one simply samples the distribution of y, computes the total entropy of that distribution and then conditions it on x. In the approach taken here, one focuses on x.
See comments above, added Supplementary figure 2 and a paragraph on this in the Materials and Methods section.

The equation for Hxx (Eq 3) seems incorrect, there should not be a minus sign between the two terms.
Indeed, there as actually a minus sign missing, corrected.

To form the mutual information, one needs the conditional distribution of x given y, and to average that over y. How is Hxy as given serving that role? The construction of the stimulus is not a process of tracking log odds, nor in reality are spike trains. The ability for the MI as written to be less than zero should be an indication that this is not quite right.
As derived in (Zeldenrust, F., de Knecht, S., Wadman, W. J., Denève, S., & Gutkin, B. S. (2017). Estimating the Information Extracted by a Single Spiking Neuron from a Continuous Input Time Series. *Frontiers in Computational Neuroscience*, *11*, 49. https://doi.org/10.3389/fncom.2017.00049), equations (8-11), the conditional entropy can be estimated from the log-odds ratio by averaging over time, assuming ergodic Poisson processes. We added a more explicit explanation. The approximation fails for very low firing rates, as explained in the methods section.

It does not make sense to normalize the firing rate by the switching rate. As different neurons have different internal dynamics, they will be affected differently by the rate of changes in the drive. In the paper, the switching rate is set to be different for different neurons and the change in switching rate is "offset" by changing the frequency of drive in the input. These things are not conceptually related and do not seem to be interchangeable, for the reasons discussed above: they change the time-averaged discriminability of the responses in different ways. The input rate changes the variance of the signal while the switching rate changes the influence of transients.
How informative a spike train (whether from the network of Poisson neuron or of a recorded neuron is) is about a binary state, depends on the number of state switches relative to the number of spikes: ideally there would be one or a few spikes per 'on state', and not many more (efficiency). Therefore, a fast-switching hidden state is more difficult to track with fewer spikes, and it is the number of spikes relative to the number of 'on-states' in a given time window (see figure 2B) that determines the amount of information that can be transmitted. Excitatory neurons in the barrel cortex respond with very low firing rates, irrespective of the stimulus properties (both for noisy stimuli as presented here, and for step-and-hold stimuli, as shown in figure 1 and also in vivo: compare https://pubmed.ncbi.nlm.nih.gov/15064767/ and https://pubmed.ncbi.nlm.nih.gov/32027647/ or for a review see https://pubmed.ncbi.nlm.nih.gov/22579264/, we added this to the discussion for clarity). It does not make sense to present a fast-fluctuating hidden state to a neuron that responds with only a low firing rate, as the mutual information between stimulus and output firing rate will always be 0. We can, however, give a slowly switching stimulus to a neuron that responds with a relatively high firing rate (which we have done as a control, see figure 3 and 4, green markers). Therefore, we are convinced that it is the number of spikes 'available' in a spike train (ie the firing rate) relative to the switching speed of the hidden state (ie the number of 'on' and 'off' states in a given time window) is the correct quantity to compare. However, we see the point that the reviewer is making about the input current. In Supplementary figure 2, N and O, we compare the Kullback-Leibler divergence between the input, membrane potential and instantaneous firing rate when x=0 and when x=1. It turns out that the Kullback-Leibler divergence between the histograms for the two states is for the membrane larger than that of the input, but then smaller again for the firing rate (something we have observed before: Huang, Chao, Bernhard Englitz, Andrey Reznik, Fleur Zeldenrust, and Tansu Celikel. 2020. "Information Transfer and Recovery for the Sense of Touch." *bioRxiv*, December, 2020.12.08.415729. https://doi.org/10.1101/2020.12.08.415729). We also see that indeed the Kullback-Leibler divergence is (as expected) smaller for the input current given to the excitatory neurons than for the inhibitory neurons (which is exactly the 'trade-off' of the method presented also in fig. 2B: a longer integration time window in exchange for a larger difference in input amplitude). Nevertheless, the information content of the spike trains does not appear to be affected much by which method of computation we use (Supplementary figure 2M).
We added these calculations and results as supplementary figure 2, and added a summary of the arguments above to the methods. We also added a paragraph in the discussion about the different type of roles a neuron could have (ie integrator, resonator or differentiator), and that our setup basically assumes neurons are integrators. We hope that with this more explicit discussion of the limitations of our method and its comparison to the other calculation the reviewer suggested, it is now more clear what our aim was and how it can be interpreted.

The data on STA forms and their variability in different neuron classes are interesting and should be better contextualized relative to or integrated with the primary focus of the paper.
We thank the reviewer for this suggestion. We have added a paragraph discussing this figure to the discussion.
